# Electrochemical Coupling of Biomass‐Derived Acids: New C_8_ Platforms for Renewable Polymers and Fuels

**DOI:** 10.1002/cssc.201601271

**Published:** 2016-12-22

**Authors:** Linglin Wu, Mark Mascal, Thomas J. Farmer, Sacha Pérocheau Arnaud, Maria‐Angelica Wong Chang

**Affiliations:** ^1^ Department of ChemistryUniversity of California Davis1 Shields AvenueDavisCA95616USA; ^2^ Green Chemistry Centre of Excellence, Department of ChemistryUniversity of YorkHeslingtonYorkYO10 5DDUK

**Keywords:** biomass conversion, electrolysis, itaconic acid, kolbe coupling, levulinic acid

## Abstract

Electrolysis of biomass‐derived carbonyl compounds is an alternative to condensation chemistry for supplying products with chain length >C_6_ for biofuels and renewable materials production. Kolbe coupling of biomass‐derived levulinic acid is used to obtain 2,7‐octanedione, a new platform molecule only two low process‐intensity steps removed from raw biomass. Hydrogenation to 2,7‐octanediol provides a chiral secondary diol largely unknown to polymer chemistry, whereas intramolecular aldol condensation followed by hydrogenation yields branched cycloalkanes suitable for use as high‐octane, cellulosic gasoline. Analogous electrolysis of an itaconic acid‐derived methylsuccinic monoester yields a chiral 2,5‐dimethyladipic acid diester, another underutilized monomer owing to lack of availability.

## Introduction

Biofuels and renewable polymers play an undisputed role in the green technology movement, and their production from biomass‐derived carbohydrates has been investigated by multiple research groups. In the case of biofuels, since common monosaccharides are C_6_ or less, C−C coupling steps are essential to eventually achieve the hydrocarbon volatility range required for automotive fuels. In the case of polymers, virtually any platform molecule that can be rendered bifunctional may potentially serve as a monomer, and C−C coupling reactions can also be of value here, likewise offering products not limited to six carbons.

**Scheme 1:**
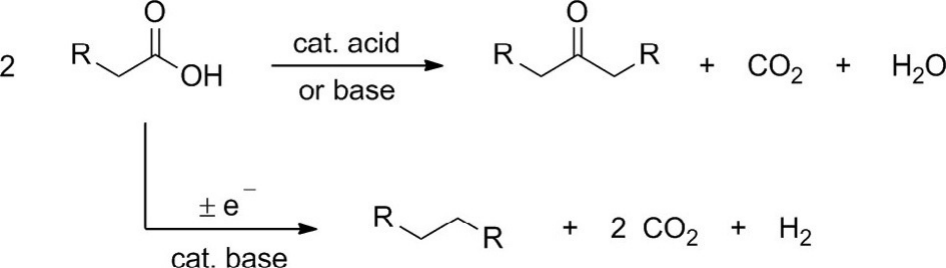
Comparison of products derived by a condensation reaction versus electrochemical coupling. Claisen chemistry, while feasible with carboxylic acids,^2^ is routinely carried out with the corresponding esters, in which case the condensate is an alcohol instead of water.

In the majority of cases, the approach to accessing suitable biofuel precursors from biomass‐derived carbonyl compounds involves aldol or related condensation reactions, which are catalyzed, thermodynamically driven processes resulting in C−C‐bond formation.^1^ We however became attracted to the proposition of carrying out electrochemical coupling of such molecules to accomplish the necessary chain elongation for the following reasons: i) the electrochemical dimerization of sugar derivatives is an inexpensive, non process‐intensive method where the driving force for the reaction essentially comes from the power grid, ii) novel structures may be accessed that are not available via condensation chemistry, and iii) coupled products can be obtained in a more advanced state of reduction, thus avoiding extensive hydrodeoxygenation. Comparison of a condensation reaction versus the Kolbe electrolysis serves as illustration (Scheme 1). Using the same substituted acetic acid as a hypothetical model, Claisen‐type condensation/decarboxylation results in a ketone, whereas electrolysis gives a substituted ethane. In both cases, oxygen is carried away in the form of CO_2_, although in the latter H_2_ is cogenerated, reminiscent of the production of H_2_ and CO_2_ from hydrocarbons by a combination of steam reforming and the water‐gas shift reaction.

Herein, we demonstrate that electrolysis of biomass‐derived carbonyl compounds has disruptive potential to deliver >C_6_ biorefinery outputs of value both in materials and fuels markets.

## Results and Discussion

**Scheme 2:**
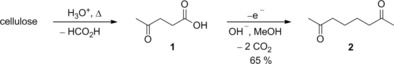
Conversion of cellulose to 2,7‐octanedione (**2**) via levulinic acid (**1**).

Looking to the literature, there has been little activity around the direct coupling of sugars, although it was interesting to find that glucose itself has been made to undergo a cathodic process whereby it was transformed into a dodecitol, presumably via its aldehyde form.^3^ The electrolysis however of sugar derivatives presents a viable alternative, and we were drawn to reports of the dimerization of levulinic acid (**1**) to 2,7‐octanedione (**2**) (Scheme 2).^4^, ^5^, ^6^ The recent upsurge of interest in **1** as a renewable, carbohydrate‐derived platform molecule, and the continuing development of technologies to produce it on an industrial scale,^7^ advance dione **2** as a potential “secondgeneration” platform only two steps removed from biomass. The electrolysis reaction is straightforward, providing 65 % yield of **2** at 90 % conversion using platinum plate electrodes in an undivided cell under constant‐current conditions (see Supporting Information for details).

**Scheme 3:**
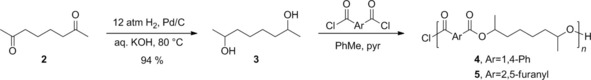
Preparation of 2,7‐octanediol (**3**) and its polyesters.

The only substantive account of the use of **2** in materials applications was published by Joshi and Limaye, who in the 1980s reported the conversion of **2** into 2,7‐octanediamine by reduction of the corresponding dioxime and subsequent production of a terephthalate polymer.^8^ Another straightforward approach to monomer synthesis would be to simply reduce **2** to the corresponding diol (2,7‐octanediol, **3**). Because of the (historically) limited access to **2**, diol **3** is largely unknown to polymer chemistry, the only reference to our knowledge being its use as one of a series of diols to test the concept of iterative tandem catalysis by polymerization with adipic esters.^9^ In that case, **3** was prepared via the corresponding α,ω‐diene. It has also been previously made from **2** using Meerwein– Pondorff–Verley reduction.^10^ However, we opted for more industrially relevant hydrogenation, which proceeded smoothly and gave **3** in high (94 %) yield (Scheme 3).

While a range of bioderived diols have been investigated for polyester synthesis, both via chemo‐ and enzyme‐catalyzed reactions, most are primary diols, typically 1,3‐propanediol, 1,4‐butanediol, and 1,6‐hexanediol.^11^ Polymerization of secondary alcohols and high molecular weight monomers is known to be more challenging owing to steric issues and high boiling points, respectively. In the standard esterification/transesterification procedure for polyester synthesis, the diol is generally used in excess relative to the diacid or diester component and high temperatures are applied to remove the excess diol and drive the polymer to high chain lengths. Attempts have been made to circumvent both the reactivity and volatility issues by using diacid chlorides in a 1:1 stoichiometric ratio with the diol.^12^ In the first instance, we applied this approach to reaction of terephthaloyl chloride with diol **3** to obtain a 63 % yield of poly(2,7‐octanediol)terephthalate (**4**), which was found to have a good chain length (*M*_n_>8500 Da) and low polydispersity index (PDI<1.5). Another motivation for using diacid chlorides was our facile, two‐step preparation of 2,5‐furandicarbonyl chloride (FDCC) from 5‐(chloromethyl)furfural (CMF), which itself is one step removed from raw biomass.^13^ Since levulinic acid **1**, the precursor to **3** (via **2**), is also a single step from CMF,^14^ this provides us an opportunity to showcase the synthesis of a new polyester with both monomers ultimately derived from a single platform molecule. The resultant poly(2,7‐octanediol)‐2,5‐furanoate polymer (**5**) from the reaction between FDCC and diol **3** was also isolated in good yield (54 %) and with a reasonable chain length and low PDI. The data for these polymers are presented in Table 1.

**Table 1:** Copolyesters of diol **3**.

Polymer	Ar	Recovery [%]	*M*_n_^[a]^ [Da]	*M*_w_^[b]^ [Da]	PDI^[c]^
**4**	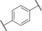	63	8531	12 220	1.43
**5**	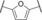	54	3978	5079	1.28

[a] Number average molecular weight. [b] Weight average molecular weight. [c] Polydispersity index (*M*_w_ / *M*_n_).

Thermogravimetric analysis under an N_2_ atmosphere was used to determine the stability of **4** and **5** (Table 2), which showed that little mass loss occurs in either prior to 290 °C. The terephthalate polyester **4** was shown to have a slightly higher thermal stability than furandioate **5**, with both its temperature at 10 and 50 % mass loss (TD10 and TD50) being roughly 20 °C higher than the furan equivalent. Likewise, the glass‐transition temperature (*T*_g_) of **4** was higher than that of **5**. In comparison to literature values for analogous aromatic–aliphatic polyesters (Table S3 in the Supporting Information), it can be seen that both **4** and **5** conform to the previously observed trend whereby extending the chain length of the diol reduces the *T*_g_ while diols of secondary alcohols show increased *T*_g_ relative to their primary alcohol regioisomers. This highlights the potential value of **3** as a new biobased monomer that allows further control of characteristics that have a direct impact on the processability and applications of the final product. For example, the near roomtemperature *T*_g_ of **5** may point to potential thermoresponsive polymer applications where changes from the glassy to rubbery state of a plastic between ambient and body temperature is desired.

**Table 2:** Thermal analysis of polymers **4** and **5**.

Polymer	TD10^[a]^ [°C]	TD50^[b]^ [°C]	*T*_g_^[c]^ [°C]	*T*_m_^[d]^
**4**	312.4	330.5	62.6	n.d.
**5**	289.5	301.6	26.0	n.d.

[a] Temperature at 10 % mass loss. [b] Temperature at 50 % mass loss. [c] Glass transition temperature as determined by differential scanning calorimetry (DSC). [d] Melting point as determined by DSC, n.d.=none detected.

**Scheme 4:**
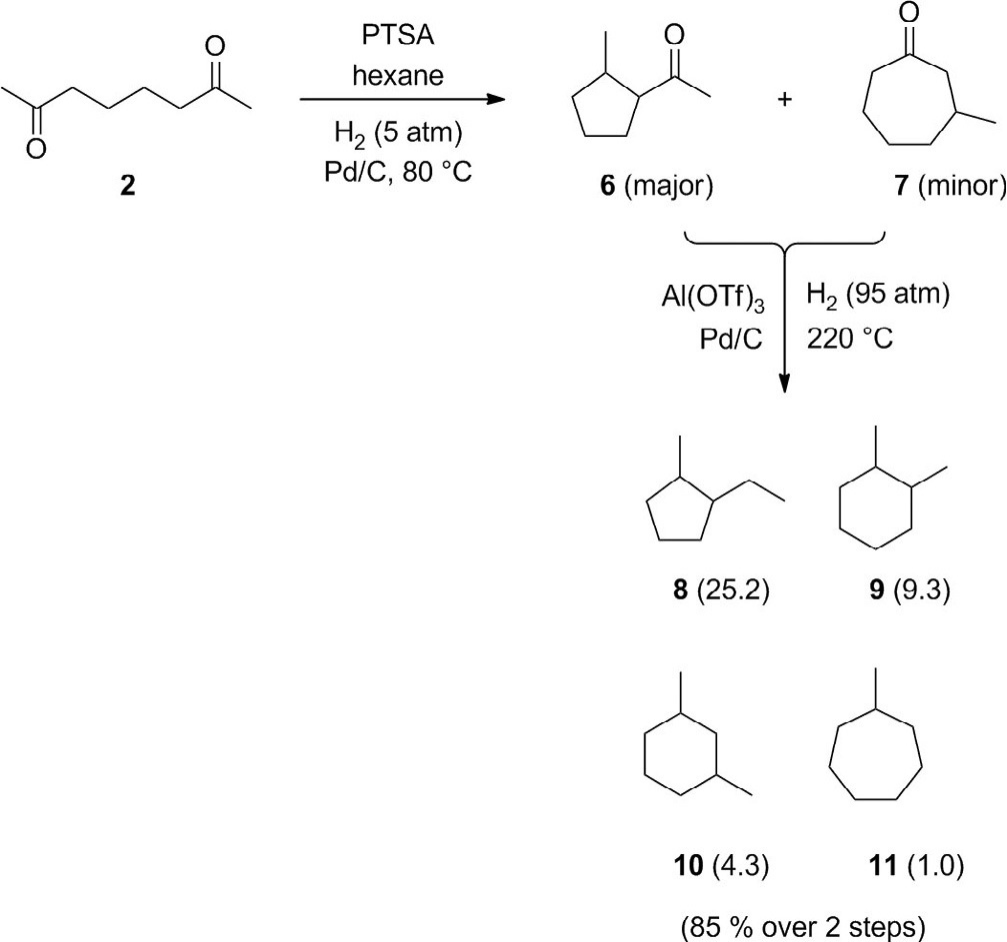
Production of cycloalkane biofuels from **2**. PTSA = *p*‐toluenesulfonic acid.

To date, the only application of **2** directed toward biofuels involved complete reduction to *n*‐octane,^15^, ^16^ a compound of no use in gasoline (research octane number, RON=−19) and too volatile for diesel fuel.^17^ We recognized an opportunity to use dione **2** to much better advantage in the production of fuels by employing intramolecular condensation chemistry leading ultimately to branched, cyclic alkanes. Thus, **2** could be made to undergo aldol condensation by treatment with either acid or base. The α,β‐unsaturated ketone product mixture was however prone to side reactions under these conditions, which made it difficult to achieve good selectivity, a result also noted by Bouillon et al.^18^ We therefore performed the aldol reaction in tandem with hydrogenation of the double bond to provide mainly methyl 2‐methylcyclopentyl ketone (**6**) alongside small amounts of the alternative cyclization product 3‐methylcycloheptanone (**7**, Scheme 4). This product mixture could be isolated and characterized, but the best yield of hydrocarbon was obtained by introducing additional Pd/C and the hydrodeoxygenation catalyst Al(OTf)_3_ 19 at this point, then increasing the reaction temperature and H_2_ pressure to 220 °C and 50 bar, respectively. Using this approach, the total yield of cycloalkanes **8**–**11** starting from **2** was 85 %, with the relative ratios as determined by GC–MS shown. The observation of dimethylcyclohexanes **9** and **10** is the result of carbocation rearrangements, and since commercial gasoline is a mixture of hundreds of hydrocarbons,^20^ the lack of selectivity to **8** presents no problems.

Since we propose the conversion of **2** to **8**–**11** as a new approach to cellulosic gasoline, we undertook to determine the fuel properties of these products. Existing biomass‐to‐biofuel processes generally target linear hydrocarbons appropriate to diesel or jet fuel applications.^1^ Efficient renewable methods that lead to the branched and cycloalkanes found in gasoline therefore fill a commercial void.^21^ **8**–**11** are all previously described compounds with boiling points between 118–134 °C, thus well within the volatility range of motor gasoline (ca. 40–200 °C).^20^ The key measure of fuel performance in spark ignition engines is the antiknock index, generally referred to as octane number. For individual molecules, a value for the RON will either have been measured or can be calculated using various algorithms. The experimental RON value for **9** is 69 and for **10** is 81.^22^ A modeled value of 93 has been reported for **8**.^23^ As for the minor component **11**, the RON of cycloheptane itself is 38.8. Methyl branching generally increases octane numbers, and the index value for this molecule has been calculated at RON=68 using the model of Dahmen and Marquardt.^24^ An estimated RON of the blend of **8**–**11** in the proportions observed in the hydrogenation reaction would be 86.8. In combination with 10 % ethanol as an oxygenate, the calculated RON is 89.

**Scheme 5:**
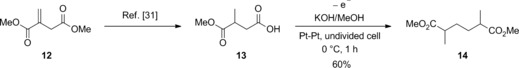
Production of dimethyl 2,5‐dimethyladipate (**14**) by electrolysis of itaconate‐derived methylsuccinic acid monoester (**13**).

Finally, our success in processing levulinic acid into useful biofuel and polymer components prompted us to consider the electrochemical dimerization of other bioderived acids. Itaconic acid (**12,** Scheme 5) is another up and coming biorefinery platform molecule,^25^ and its applications to heterocycle and polymer chemistry have recently been reviewed.^26^, ^27^ Like **1**, it appears on the National Renewable Energy Laboratory (NREL) list of the top 12 value‐added chemicals from biomass.^28^ Although the electrolysis of **12** has been investigated,^29^ no Kolbe coupling of the acid itself or its monoesters has been reported to date. However, Hancock and Linstead showed that a 2‐methylsuccinic acid 1‐methyl ester (**13**), derived by the methanolysis of methylsuccinic anhydride, underwent electrolytic dimerization to dimethyl 2,5‐dimethyladipate (**14**).^30^ In that work, a poorly defined mixture of half‐esters was used and the reported yield of **14** was 30 %. We set out to improve access to **14** by optimization of the electrochemical reaction. Thus, ester **13** was prepared from itaconic acid **12** using a published method, wherein the diester of **12** was selectively hydrolyzed and the double bond asymmetrically hydrogenated (Scheme 5).^31^ While a stereodefined form of **13** presents an attractive option for future work, for the purposes of this study, the racemate was used. Subjecting **13** to the same conditions as in the conversion of **1** to **2**, that is, constant current electrolysis at a current density of 180 mA cm^−2^ across platinum electrodes in an undivided cell, gave diester **14** in 60 % yield at 85 % conversion.

Despite being a simple analogue of one of the highest volume monomers used in industry, limited access has meant that very little in the way of polymer chemistry has been described for **14**, as was also the case for **3**. The only systematic study of materials derived from **14** involved the production of stereoisomeric polyamides from hexamethylene diamine and *meso*‐, *d*‐, and *dl*‐**14**, all of which showed considerably less crystallinity than the parent polymer.^32^ The development of new applications for **14** would be stimulated by increased availability. Of particular interest would be a study of the properties of polyesters of **3** with **14**, and we will report on such materials in a separate paper.

## Conclusions

The purpose of this work is to showcase the opportunities for accessing new platform molecules by electrochemical processing of primary biomass derivatives. The diversity of >C_6_ structures attainable using this approach points to future materials and fuel markets comprising a range of novel products. Here, we have presented levulinic acid (**1**) as a precursor to branched C_8_ monomers and cycloalkane components that embody a high‐octane, cellulosic gasoline, both via 2,7‐octanedione (**2**). The monomer 2,7‐octanediol (**3**) was used to prepare new polyesters with terephthalic and 2,5‐furandicarboxylic acids. An analogous renewable monomer synthesis was demonstrated in dimethyl 2,5‐dimethyladipate (**14**). Novel access to **3** and **14** by means of electrochemical C−C‐coupling chemistry opens up new opportunities for products that were formerly both limited in their availability and produced from unsustainable feeds.

## Experimental Section

### Electrolysis of levulinic acid

Levulinic acid (464 mg, 4.00 mmol) was subjected to constant current electrolysis (178 mA cm^−2^) on platinum plate electrodes (1.5×1.5 cm^2^; distance between electrodes=12 mm) in methanolic KOH (10 mL, 0.075 m) using an undivided cell with magnetic stirring at 22 °C. The reaction was terminated after the consumption of 1.0 F mol^−1^ of charge (16 min). The mixture was acidified to pH 3 using 1 m HCl and the volatiles were evaporated under vacuum. The conversion (90 %) and yield of **2** (65 %) were determined by NMR spectroscopy using 1,4‐dioxane as internal standard. NaOH (50 mL, 1 m) was added to the residue and the mixture was extracted with dichloromethane (50 mL×3). The combined organic extract was washed with brine and dried over sodium sulfate. The solution was filtered and concentrated under vacuum, and the residue was passed through a short plug of silica gel using ethyl acetate as eluent. The solvent was evaporated to give **2** as a pale yellow solid (178 mg, 63 %). ^1^H NMR (600 MHz, CDCl_3_): *δ*=2.44–2.42 (m, 4 H), 2.12 (s, 6 H), 1.55–1.53 ppm (m, 4 H); ^13^C NMR (150 MHz, CDCl_3_): *δ*=208.63, 43.38, 29.90, 23.14 ppm.

### Hydrogenation of 2,7‐octanedione

2,7‐Octanedione (994 mg, 6.99 mmol), Pd/C (280 mg, 5 %), KOH (140 mg, 2.1 mmol), and water (35 mL) were introduced into a Parr hydrogenator. The vessel was sealed, flushed three times with H_2_ and pressurized to 12 bar. The mixture was heated at 80 °C with stirring for 140 min, then allowed to cool to room temperature. The pressure was released and the reaction was filtered through a short pad of Celite. The filtrate was concentrated under vacuum and the residue was filtered through a short plug of silica gel using acetone as eluent. Evaporation of the solvent gave **3** as a colorless oil (962 mg, 94 %). ^1^H NMR (400 MHz, D_2_O): *δ*=3.78–3.71 (m, 2 H), 1.39–1.26 (m, 8 H), 1.08 ppm (d, *J=*6.3 Hz, 3 H); ^13^C NMR (100 MHz, D_2_O): *δ*=67.87, 37.78, 24.84, 21.82 ppm.

### Tandem intramolecular aldol condensation and catalytic hydrogenation of 2,7‐octanedione

2,7‐Octanedione (284 mg, 2.00 mmol), Pd/C (42 mg, 10 %), *p*‐toluenesulfonic acid monohydrate (38 mg), and hexanes (10 mL) were introduced into a Parr hydrogenator. The vessel was sealed, flushed three times with H_2_, and pressurized to 5 bar. The reaction was heated at 80 °C with stirring for 3.5 h and allowed to cool to room temperature, followed by further cooling to 10 °C in an ice‐water bath. The reaction mixture was filtered through silica gel and further eluted with an ethyl acetate/hexane (1:5) solvent mixture. Evaporation of the solvent resulted in a mixture of isomeric ketones **6** (major) and **7** (minor) (227 mg, 90 %). The above reaction was repeated, but instead of isolating **6** and **7** the reactor was opened and Al(OTf)_3_ (95 mg), additional Pd/C (168 mg, 10 %), and hexanes (30 mL) were added. The vessel was sealed, flushed three times with H_2_ and pressurized to 50 bar H_2_. The reaction was heated to 220 °C, which increased the internal pressure to approximately 95 bar. After 24 h at this temperature, the reactor was allowed to cool to room temperature and then further to 0 °C in an ice‐water bath. The interior walls of the vessel were washed down with acetone. The catalyst was removed by filtration through Celite and the yields of **8**–**11** (85 % total) were determined by GC–MS analysis with a dodecane internal standard and data matching against the National Institute of Standards and Technology (NIST) mass‐spectral library.

1‐Ethyl‐2‐methylcyclopentane (**8**): EIMS: *m*/*z* (% of max intensity) 41.2 (58), 55.1 (100), 70.2 (55), 83.2 (95), 97.2 (11), 112.1 (24); retention time in GC–MS: 3.42 (*trans*) and 3.85 min (*cis*); *trans*/*cis* ratio is 2.1:1.

1,3‐Dimethylcyclohexane (**9**): EIMS: *m*/*z* (% of max intensity) 41.2 (21), 55.2 (70), 69.2 (19), 97.2 (100), 112.1 (31); retention time in GC–MS: 3.26 (*trans*) and 3.62 min (*cis*); *trans*/*cis* ratio is 1.8:1.

1,2‐Dimethylcyclohexane (**10**): EIMS: *m*/*z* (% of max intensity) 41.1 (40), 55.2 (91), 70.2 (27), 83.1 (20), 97.2(100), 112.2 (36); retention time in GC–MS: 3.54 (*trans*) and 3.97 min (*cis*); *trans*/*cis* ratio is 2.3:1.

Methylcycloheptane (**11**): EIMS: *m*/*z* (% of max intensity) 41.1 (71), 55.1 (98), 69.1 (35), 83.1 (40), 97.2(100), 112.1 (20); retention time in GC–MS: 4.37 min.

### Electrolysis of 2‐methylsuccinic acid 1‐methyl ester

2‐Methylsuccinic acid 1‐methyl ester (**13**) (1.17 g, 8.01 mmol) was subjected to constant‐current electrolysis (178 mA cm^−2^) on platinum plate electrodes (1.5×1.5 cm^2^; distance between electrodes=12 mm) in methanolic KOH (20 mL, 0.10 m) using an undivided cell with magnetic stirring at 0 °C. The reaction was terminated after the consumption of 2.0 F mol^−1^ of charge (64 min). A small sample of the reaction was acidified to pH 3 using 1 m HCl and evaporated under vacuum. The conversion was determined by NMR spectroscopy (85 %) using 1,4‐dioxane as internal standard. The reaction mixture was evaporated under vacuum and NaOH (70 mL, 0.1 m) was added to the residue. The solution was extracted with dichloromethane and the combined organic extract was washed with saturated brine and dried over sodium sulfate. The solution was filtered and the solvent was evaporated to give dimethyl 2,5‐dimethyladipate (**14**) as a colorless oil (489 mg, 60 %). ^1^H NMR (400 MHz, CDCl_3_): *δ*=3.64 (s, 6 H), 2.43–2.37 (m, 2 H), 1.65–1.56 (m, 2 H), 1.44–1.32 (m, 2 H), 1.12 ppm (d, *J=*7.0 Hz, 6 H); ^13^C NMR (100 MHz, CDCl_3_): *δ*=176.81, 176.75, 51.47, 39.37, 39.20, 31.32, 31.08, 17.06, 16.89 ppm.

Experimental details for the preparation of polyesters **4** and **5** are provided in the Supporting Information.

All data used in the preparation of this manuscript for the sections funded by the EPSRC grant EP/L017393/1 is contained within this document, the Supporting Information, or can be requested from from https://doi.org/10.15124/93ff2cd7‐9408‐4c34‐aac6‐3baa4f50abff.

## Supporting information

As a service to our authors and readers, this journal provides supporting information supplied by the authors. Such materials are peer reviewed and may be re‐organized for online delivery, but are not copy‐edited or typeset. Technical support issues arising from supporting information (other than missing files) should be addressed to the authors.

Supplementary

## References

[1] L. Wu , T. Moteki , A. A. Gokhale , D. W. Flaherty , F. D. Toste , Chem 2016, 1, 32–58.

[2] For an example of a metal-oxide catalyzed version of this reaction, see: L. M. Orozco , M. Renz , A. Corma , ChemSusChem 2016, 9, 2430–2442. 27539722 10.1002/cssc.201600654

[3] M. L. Wolfrom , W. W. Binkley , C. C. Spencer , B. W. Lew , J. Am. Chem. Soc. 1951, 73, 3357–3358.

[4] H. Hofer , Ber. Dtsch. Chem. Ges. 1900, 33, 650–657.

[5] S. Shimizu , Nippon Nogei Kagaku Kaishi 1950, 23, 288–294.

[6] I. Cabasso , M. Li , Y. Yuan , RSC Adv. 2012, 2, 9998–10006.

[7] *The Biofine Process-Production of Levulinic Acid, Furfural, and Formic Acid from Lignocellulosic Feedstocks*: D. J. Hayes , S. W. Fitzpatrick , M. H. B. Hayes , J. R. H. Ross in Biorefineries-Industrial Processes and Products: Status Quo and Future Directions, Vol. 1 (Eds.: B. Kamm, P. R. Gruber, M. Kamm), Wiley-VCH, Weinheim, 2006, pp. 144–160; see also:

[8] U. R. Joshi , P. A. Limaye , Biovigyanam 1985, 11, 101–103.

[9] B. A. C. van As , J. van Buijtenen , T. Mes , A. R. A. Palmans , E. W. Meijer , Chem. Eur. J. 2007, 13, 8325–8332. 17659517 10.1002/chem.200700818

[10] U. R. Joshi , P. A. Limaye , Ind. J. Chem. B 1986, 25, 1176–1178.

[11] C. Vilela , A. F. Sousa , A. C. Fonseca , A. C. Serra , J. F. J. Coelho , C. S. R. Freire , A. J. D. Silvestre , Polym. Chem. 2014, 5, 3119–3141.

[12] M. Gomes , A. Gandini , A. J. D. Silvestre , B. Reis , J. Polym. Sci. Part A 2011, 49, 3759–3768.

[13] S. Dutta , L. Wu , M. Mascal , Green Chem. 2015, 17, 3737–3739.

[14] M. Mascal , E. B. Nikitin , Green Chem. 2010, 12, 370–373.

[15] P. Nilges , T. R. dos Santos , F. Harnisch , U. Schröder , Energy Environ. Sci. 2012, 5, 5231–5235.

[16] T. R. dos Santos , P. Nilges , W. Sauter , F. Harnisch , U. Schröder , RSC Adv. 2015, 5, 26634–26643.

[17] *Diesel Fuels Technical Review*, Chevron Corporation **2007**, http://www.chevron.com/-/media/chevron/operations/documents/diesel-fuel-tech-review.pdf.

[18] J.-P. Bouillon , C. Portella , J. Bouquant , S. Humbel , J. Org. Chem. 2000, 65, 5823–5830. 10970329 10.1021/jo005544y

[19] H.-J. Song , J. Deng , M.-S. Cui , X.-L. Li , X.-X. Liu , R. Zhu , W.-P. Wu , Y. Fu , ChemSusChem 2015, 8, 4250–4255. 26611542 10.1002/cssc.201500907

[20] *Motor Gasolines Technical Review*, Chevron Corporation **2009**, http://www.chevron.com/-/media/chevron/operations/documents/motor-gas-tech-review.pdf.

[21] M. Mascal , S. Dutta , I. Gandarias , Angew. Chem. Int. Ed. 2014, 53, 1854–1857; 10.1002/anie.201308143 24474249

[22] *Knocking characteristics of pure hydrocarbons (Knocking Characteristics of Pure Hydrocarbons*, STP225-EB, ASTM International, West Conshohocken, PA, 1958, https://doi.org/10.1520/STP225-EB.

[23] A. L. Lapidus , E. A. Smolenskii , V. M. Bavykin , T. N. Myshenkova , L. T. Kondrat′ev , Pet. Chem. 2008, 48, 277–286.

[24] M. Dahmen , W. Marquardt , Energy Fuels 2015, 29, 5781–5801.

[25] M. Okabe , D. Lies , S. Kanamasa , E. Y. Park , Appl. Microbiol. Biotechnol. 2009, 84, 597–606. 19629471 10.1007/s00253-009-2132-3

[26] A. M. Medway , J. Sperry , Green Chem. 2014, 16, 2084–2101.

[27] T. Robert , S. Friebel , Green Chem. 2016, 18, 2922–2934.

[28] *Top Value Added Chemicals From Biomass. Volume I: Results of Screening for Potential Candidates from Sugars and Synthesis Gas*, Technical report identifier PNNL-14804, Pacific Northwest National Laboratory and the National Renewable Energy Laboratory, **2004** (http://www.nrel.gov/docs/fy04osti/35523.pdf).

[29] G. Aarland , J. Prakt. Chem. 1873, 6, 256–272.

[30] J. E. H. Hancock , R. P. Linstead , J. Chem. Soc. 1953, 3490–3496.

[31] K. Achiwa , P. A. Chaloner , D. Parker , J. Organomet. Chem. 1981, 218, 249–260.

[32] J. H. Brewster , J. Am. Chem. Soc. 1951, 73, 366–370.

