# Supplementary material for: Electrochemical Coupling of Biomass‐Derived Acids: New C8 Platforms for Renewable Polymers and Fuels
Source: ChemSusChem. 2016 Dec 22;10(1):166–70. doi: 10.1002/cssc.201601271 (PMC5324636; doi:10.1002/cssc.201601271)
Supplement: Supplementary file 1 — Supplementary [file CSSC-10-166-s001.pdf]

CHEMISTRY & SUSTAINABILITY
CHEMSUSCHEM
ENERGY & MATERIALS

Supporting Information

Electrochemical Coupling of Biomass-Derived Acids: New C_8 Platforms for Renewable Polymers and Fuels

Linglin Wu,[a] Mark Mascal,*[a] Thomas J. Farmer,[b] Sacha Pérocheau Arnaud,[b] and Maria-Angelica Wong Chang[a]

cssc_201601271_sm_miscellaneous_information.pdf

Supporting Information

Electrochemical Coupling of Biomass-Derived Acids: New C_8 Platforms for Renewable Polymers and Fuels

Linglin Wu,[a] Mark Mascal,* [a] Thomas J. Farmer,[b] Sacha Pérocheau Arnaud[b] and Maria-Angelica Wong Chang[a]

[a] Dr. L. Wu, Prof. M. Mascal, Ms. M.-A. Wong Chang

Department of Chemistry
University of California Davis
1 Shields Avenue
Davis, CA 95616 (USA)
E-mail: mjmascal@ucdavis.edu

[b] Dr. T. J. Farmer, Mr. S. Pérocheau Arnaud
Green Chemistry Centre of Excellence
Department of Chemistry
University of York
Heslington, York, YO10 5DD (UK)

Contents

General	S2
Optimization of the Kolbe electrolysis of levulinic acid	S3-S4
Experimental procedures for polymers	S5-S6
NMR Spectra	S7-S14
Fuel analysis by gas chromatography	S15
Polymer analysis by DSC and TGA	S16-S17
Comparison of the glass transition temperature (T_g) of polyesters of 2,7-octanediol **3** with previously reported aliphatic-aromatic polyesters	S18
References	S18

General

All materials were used as received. Levulinic acid (98%), KOH (85%) $Al(OTf)_3$, *p*-toluenesulfonic acid monohydrate ($PTSA.H_2O$), terephthaloyl chloride, methanol, anhydrous toluene, and anhydrous pyridine were purchased from Sigma-Aldrich. Palladium on activated carbon was purchased from Strem Chemical. 2,7-Octanedione **2** was dried over molecular sieves prior to use in polymerizations.

^{1}H NMR spectra were recorded using a Bruker 600 NMR spectrometer operating at 600 MHz. ^{13}C NMR spectra were recorded on the same instrument at an operating frequency of 150 MHz. An HP 6002A DC power supply was used in the electrolysis reactions.

Gel permeation chromatography was used to determine polymer molecular weight and was carried out using a set (PSS SDV High) of three analytical columns (300 x 8mm, particle diameter 5 μm) of 1000, 10^5 and 10^6 Å pore sizes, plus guard column, supplied by Polymer Standards Service GmbH (PSS) and installed in a PSS SECcurity GPC system. Elution was done with stabilised tetrahydrofuran at 1ml/min with a column temperature of 23 °C and detection by refractive index. 20 μL of a 1 mg/mL sample in THF was injected for each measurement and eluted for 40 min. Calibration was carried out in the molecular weight range 400 – $2x10^6$ Da using ReadyCal polystyrene standards supplied by Sigma Aldrich.

Modulated differential scanning calorimetry (MDSC) experiments were carried out on a TA Instruments Q2000 under a nitrogen atmosphere at a heating rate of 10 °C/min up to a temperature of 200 °C, and using a sample mass of approximately 10 mg. The T_g values were reported from second heating scans.

Thermogravimetric analysis (TGA) was performed on a PL Thermal Sciences STA 625 thermal analyzer. About 10 mg of accurately weighed sample in an aluminum sample cup was placed into the analyzer furnace with a N_2 flow of 100 ml/min and heated from room temperature to 625 ˚C at a heating rate of 10 °C/min.

Optimization of the Kolbe electrolysis of levulinic acid

Kolbe electrolysis of levulinic acid **1** was carried out with platinum plate electrodes as working and counter electrodes (1.5 x 1.5 cm^2; distance between parallel electrodes = 12 mm) in a methanolic KOH solution in an undivided glass cell with magnetic stirring. Constant current was applied at the stated temperature for the stated periods of time (see Tables S1 and S2). The reaction mixture was acidified to pH=2-3 with 1 M aq HCl and then evaporated under vacuum. The conversion and yield were determined by NMR using 1,4-dioxane as an internal standard. To the residue was added 1M NaOH (50 mL) and the mixture was extracted with dichloromethane (50 ml x 3). The combined organic phase was washed with saturated brine and dried over Na_2SO_4. The solution was filtered through a short plug of silica gel and concentrated to give 2,7-octanedione **2** as a light yellow solid.

Table S1. Summary of experimental conditions (reaction time, levulinic acid concentration, methanolic KOH concentration, temperature, and current density) and the corresponding yields and conversions for the Kolbe electrolysis of levulinic acid carried out at 4 mmol scale.

Run	Time (min)	[LA] (M)	[KOH] (M)	T (˚C)	Current density (mA/cm^2)	Yield (%)	Conversion (%)
1	32	0.4	0.1	0	178	60	100
2	16	0.4	0.1	0	178	53	81
3	16	0.4	0.1	22	178	58	86
4	32	0.8	0.1	22	178	55	81
5	16	0.4	0.4	22	178	29	62
6	16	0.4	0.4	22	356	37	83
7	16	0.4	0.075	22	178	64	92

Table S2. Summary of experimental conditions (reaction time, levulinic acid concentration, methanolic KOH concentration, temperature, and current density) and the corresponding yields and conversions for the Kolbe electrolysis of levulinic acid carried out at 8 mmol, 16 mmol, 32, and 64 mmol scale.

Run	Scale (mmol)	Time (min)	[LA] (M)	[KOH] (M)	T (˚C)	Current density (mA/cm^2)	Yield (%)	Conversion (%)
1	8	32	0.4	0.075	22	178	56	87
2	16	64	0.4	0.075	22	178	58	86
3	32	128	0.4	0.075	22	178	56	85
4	64	256	0.4	0.075	22	178	48	84

Experimental procedures for polymers

Poly(2,7-octanediol)terephthalate 4

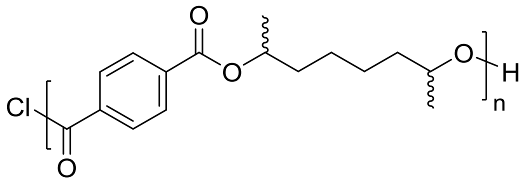

Terephthaloyl chloride (0.528 g, 2.60 mmol) was dissolved in anhydrous toluene (1.5 mL) in a dry 25 mL round-bottom flask which was sealed with the septum. The flask was cooled in an ice/water bath and continually purged with argon. Separately, 2,7-octanediol (0.380 g, 2.60 mmol) was dissolved in a mixture of anhydrous pyridine (1.7 mL) and anhydrous toluene (1.5 mL). This mixture was added via syringe to the argon purged diacid chloride solution with slow agitation by a magnetic stirrer bar. Upon mixing the reaction become cloudy and slightly viscous, with viscosity increasing over time. After 6 h the flask was removed from the ice/water bath and allowed to reach rt, being left to stir slowly. At 48 h from the start of reaction (42 hours at rt), further terephthaloyl chloride (5 mg) in anhydrous toluene (0.2 mL) was added to the reaction flask, and this was repeated again after 76 h. After a total reaction time of 92 h (86 h at rt) the excess pyridine and toluene was mostly removed *in vacuo* and the solid sticky residue was triturated twice with dry methanol (15 mL). The sticky crude product was re-dissolved in toluene (0.2 mL) and this solvent was subsequently removed under vacuum. The solid product was dried under high vacuum (<1 mbar) overnight to give poly(2,7-octanediol) terephthalate **4** as a light yellow, sticky solid (0.452 g, 63% yield based on a C.R.U. of polymer of 276.33 g mol^{-1}).

Poly(2,7-octanediol)-2,5-furanoate 5

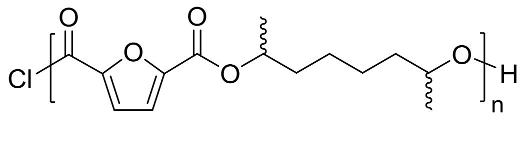

Furan-2,5-dicarbonyl chloride (0.502 g, 2.60 mmol) was dissolved in anhydrous toluene (1.5 mL) in a dry 25 mL round-bottom flask which was sealed with a septum. The flask was cooled in an ice/water bath and continually purged with argon. Separately, 2,7-octanediol (0.380 g, 2.60 mmol) was dissolved in a mixture of

anhydrous pyridine (1.7 mL) and anhydrous toluene (1.5 mL). This mixture was added via syringe to the argon purged diacid chloride solution with slow agitation by a magnetic stirrer bar. Upon mixing the reaction become cloudy and slightly viscous, with viscosity increasing over time. After 6 h the flask was removed from the ice/water bath and allowed to reach rt, being left to stir slowly. At 48 hours from the start of reaction (42 hours at rt) further terephthaloyl chloride (5 mg) in anhydrous toluene (0.2 mL) was added to the reaction flask, and this was repeated again after 76 h. After a total reaction time of 92 h (86 h at rt) the excess pyridine and toluene was mostly removed under vacuum and the solid sticky residue was triturated twice with dry methanol (15 mL). The sticky crude product was re-dissolved in 5 mL of toluene and this solvent subsequently removed under vacuum, this step was found to reduce residual pyridine in the product. The sticky crude product was re-dissolved in toluene (0.2 mL) and this solvent was subsequently removed under vacuum. The solid product was dried under high vacuum (<1 mbar) overnight to give poly(2,7-octanediol)-2,5-furanoate as an orange/yellow glassy solid (0.376 g, 54% yield based on a C.R.U. of polymer of 266.29 g mol^{-1}).

NMR spectra

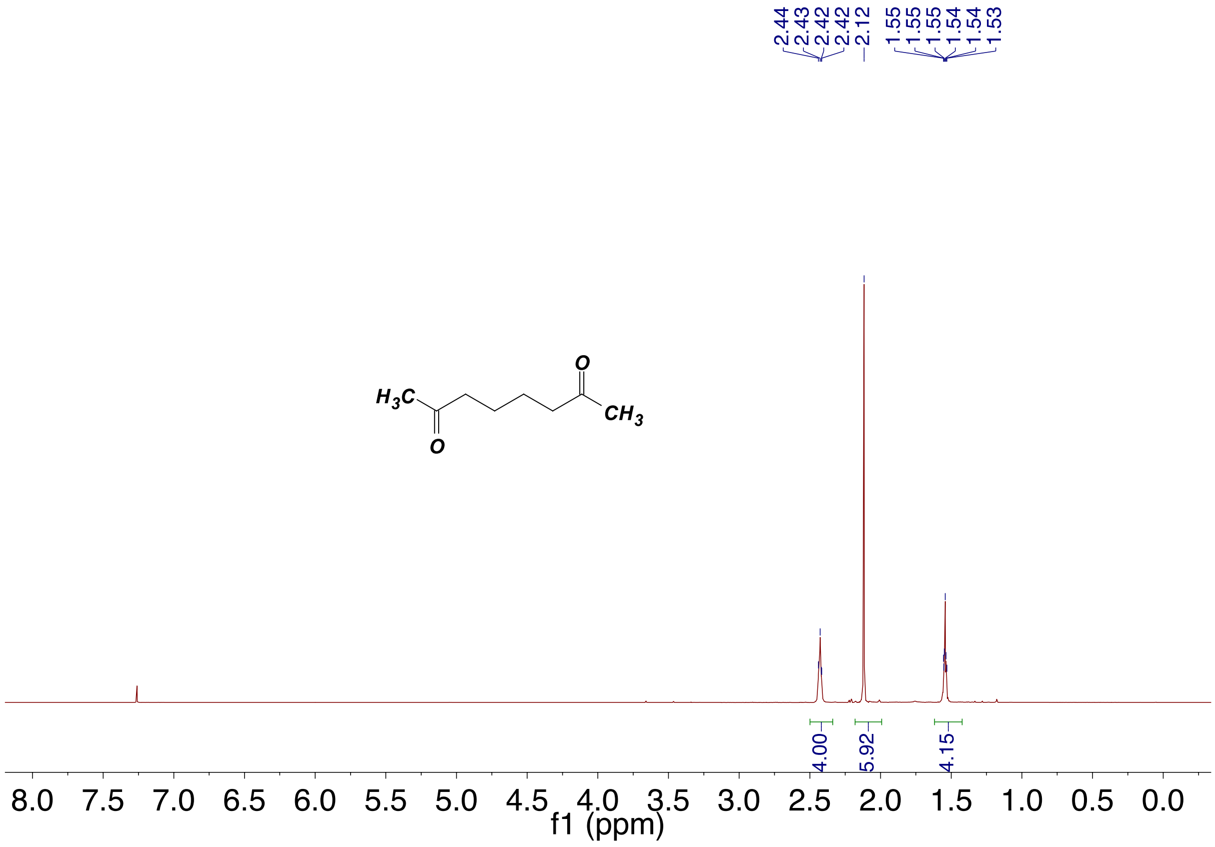

Figure S1. ^{1}H NMR spectrum of 2,7-octanedione **2** in $CDCl_3$

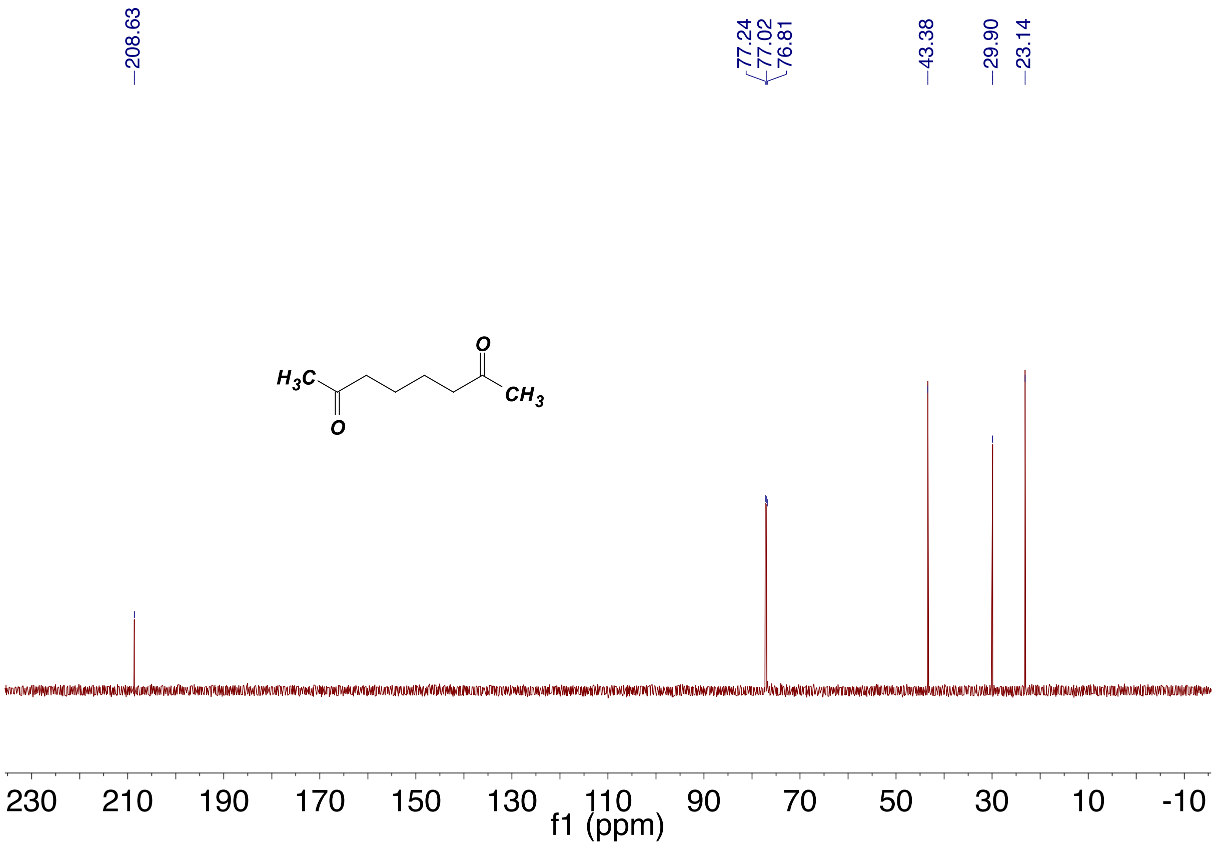

Figure S2. ^{13}C NMR spectrum of 2,7-octanedione **2** in $CDCl_3$

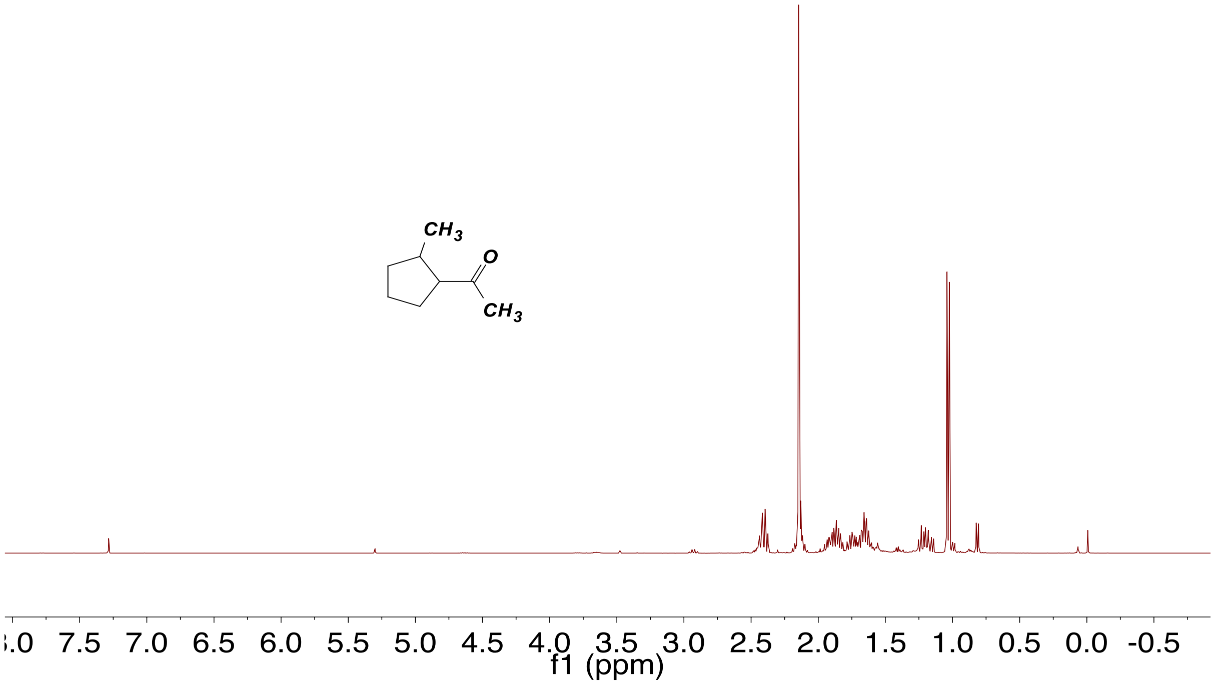

Figure S3. ^{1}H NMR spectrum of the crude product from the intramolecular aldol condensation-hydrogenation of **2** (in $CDCl_3$) (main product **6** shown).

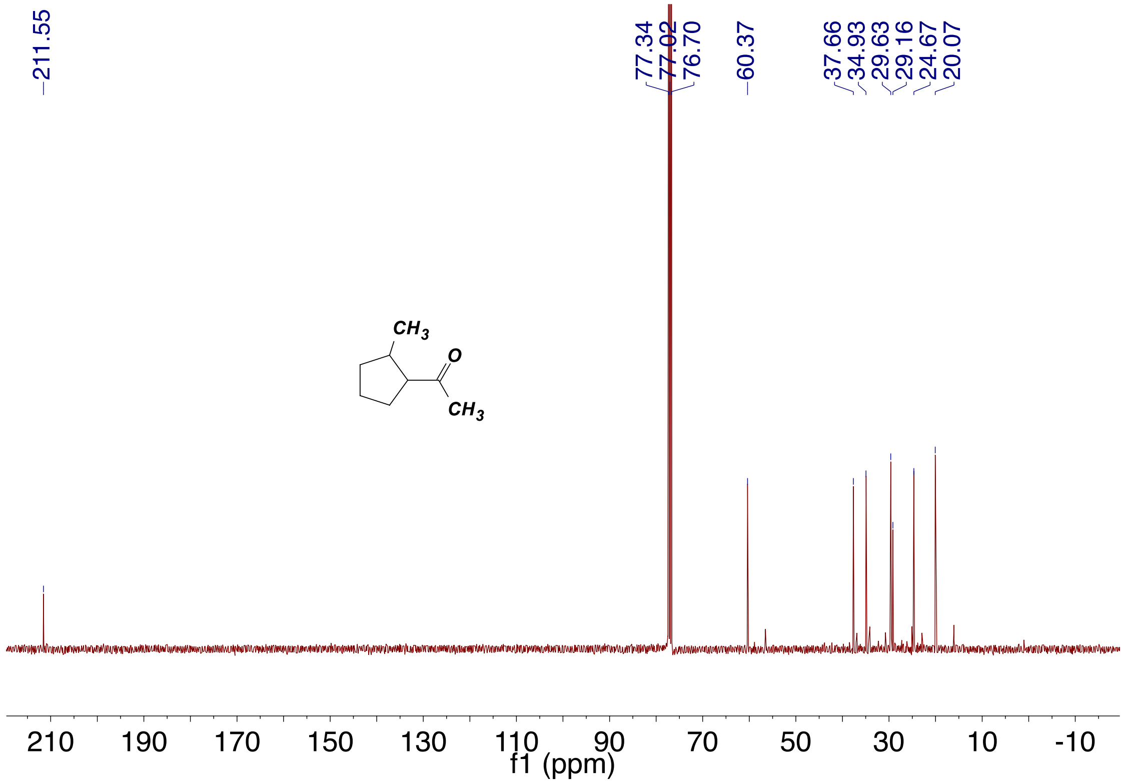

Figure S4. ^{13}C NMR spectrum of the crude product from the intramolecular aldol condensation-hydrogenation of **2** (in $CDCl_3$) (main product **6** shown).

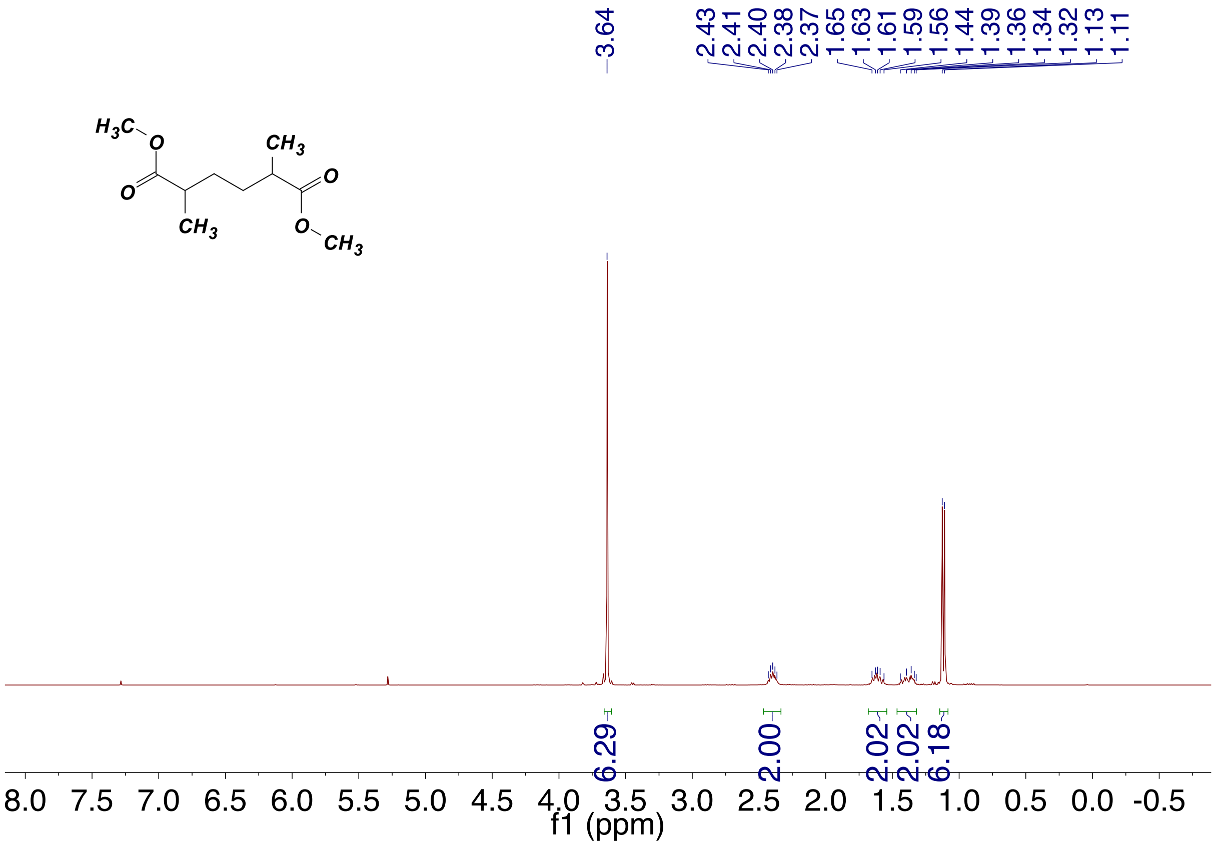

Figure S5. ^{1}H NMR spectrum of dimethyl 2,5-dimethyladipate **14** in $CDCl_3$.

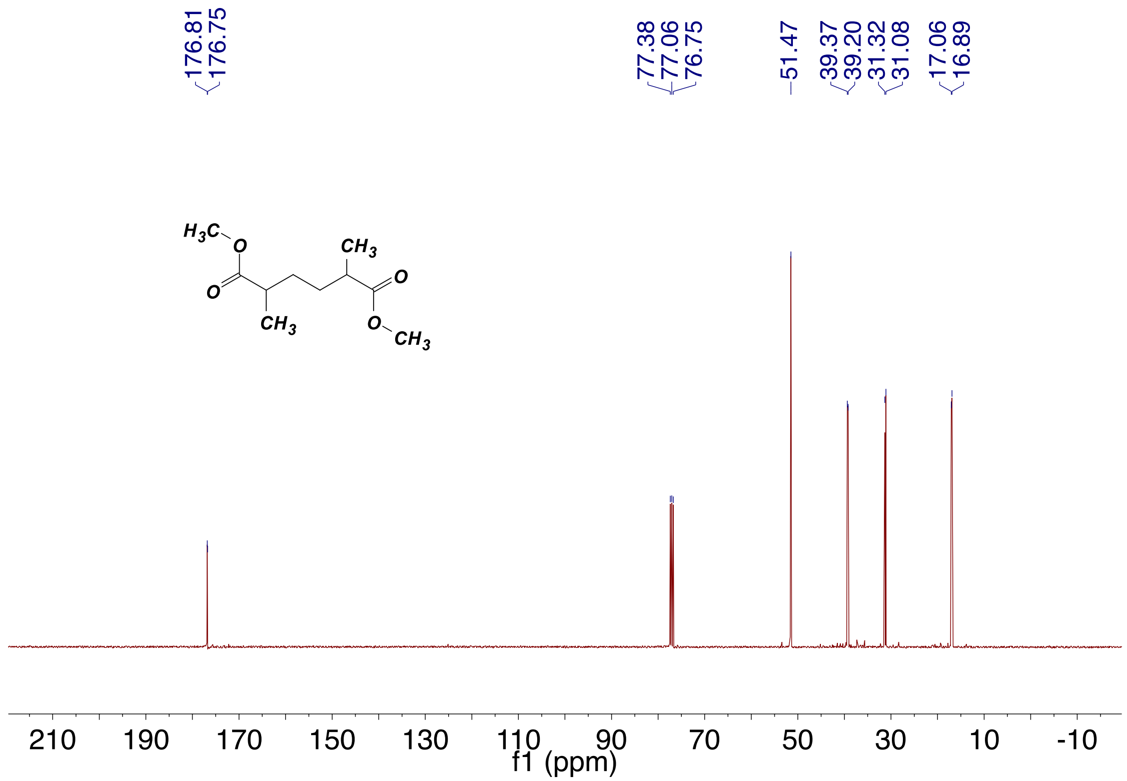

Figure S6. ^{13}C NMR spectrum of dimethyl 2,5-dimethyladipate **14** in $CDCl_3$.

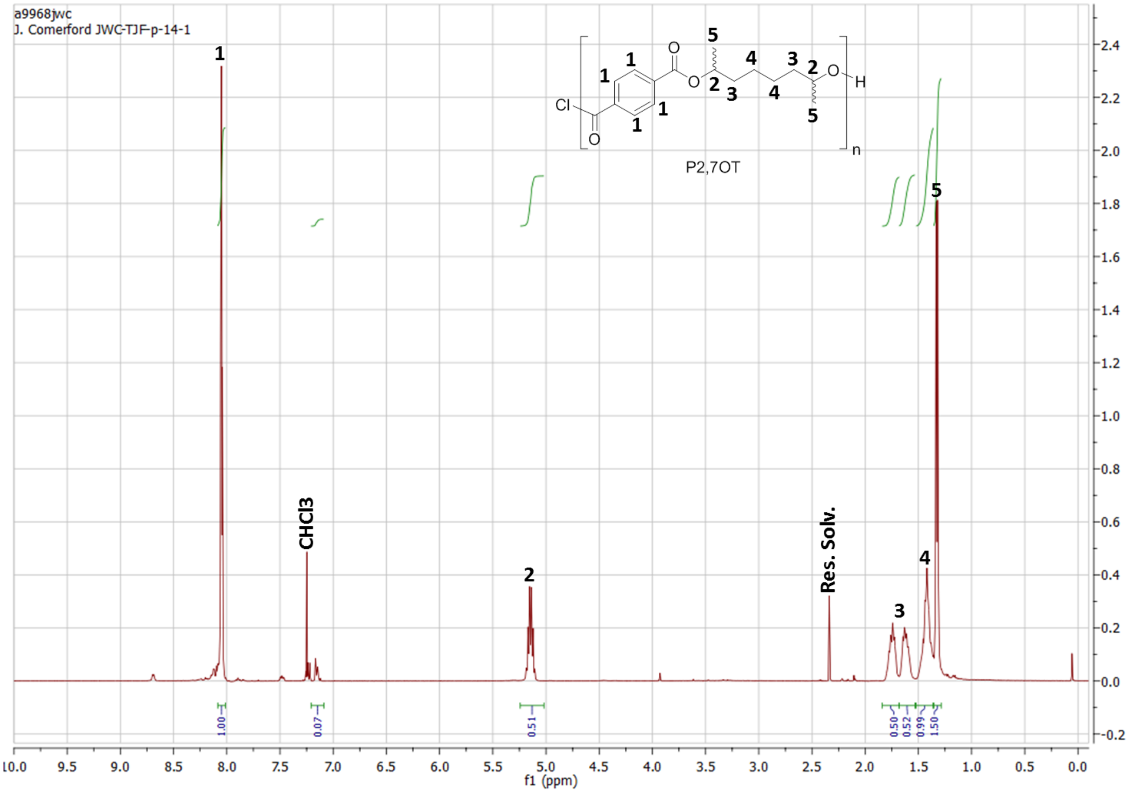

Figure S7. ^{1}H NMR spectrum of poly(2,7-octanediol)terephthalate **4**

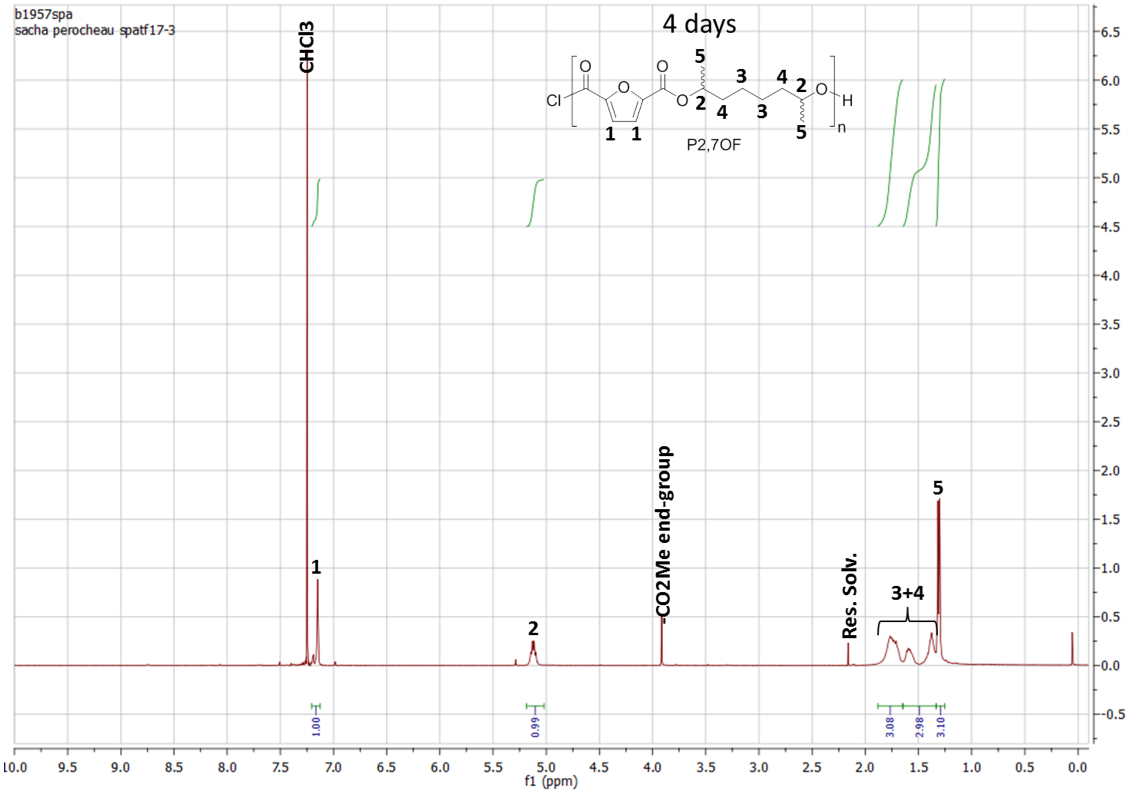

Figure S8. ^{1}H NMR spectrum of poly(2,7-octanediol)-2,5-furanoate **5**.

Fuel analysis by gas chromatography

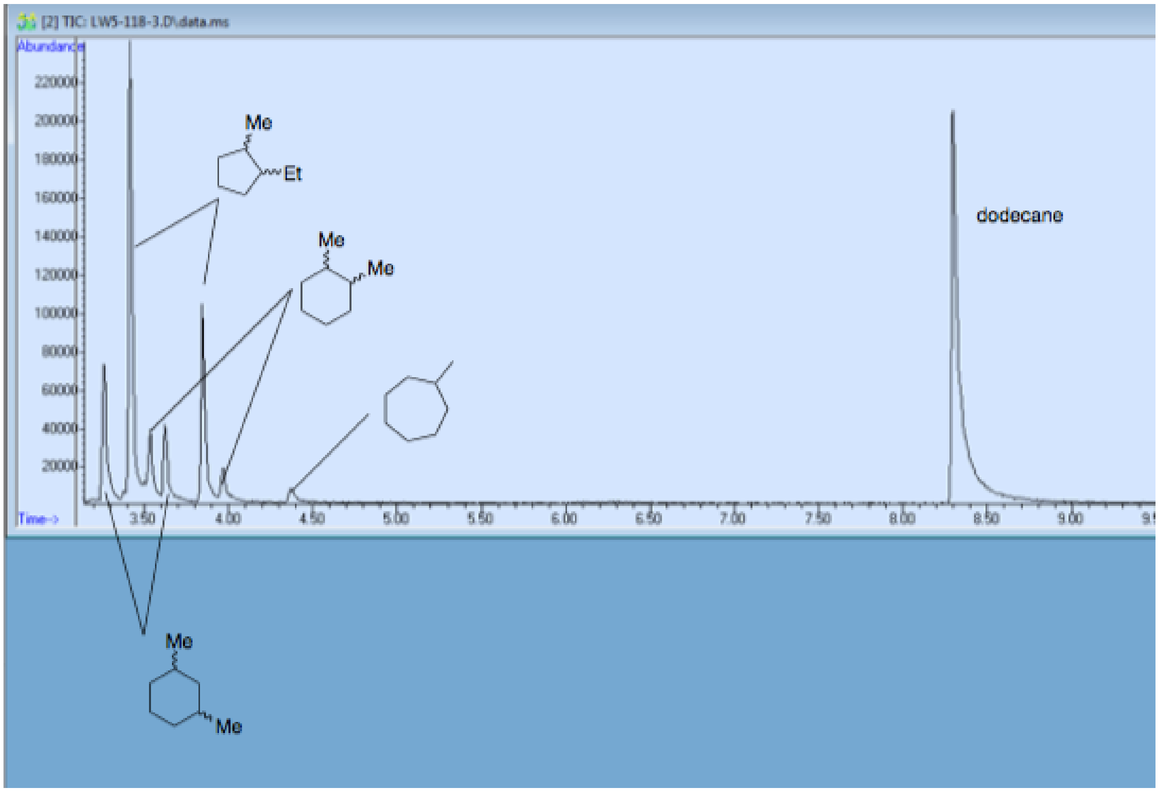

Figure S9. GC trace of the cycloalkanes resulting from the hydrogenation of **6** and **7**, with dodecane as internal standard.

Polymer analysis by DSC and TGA

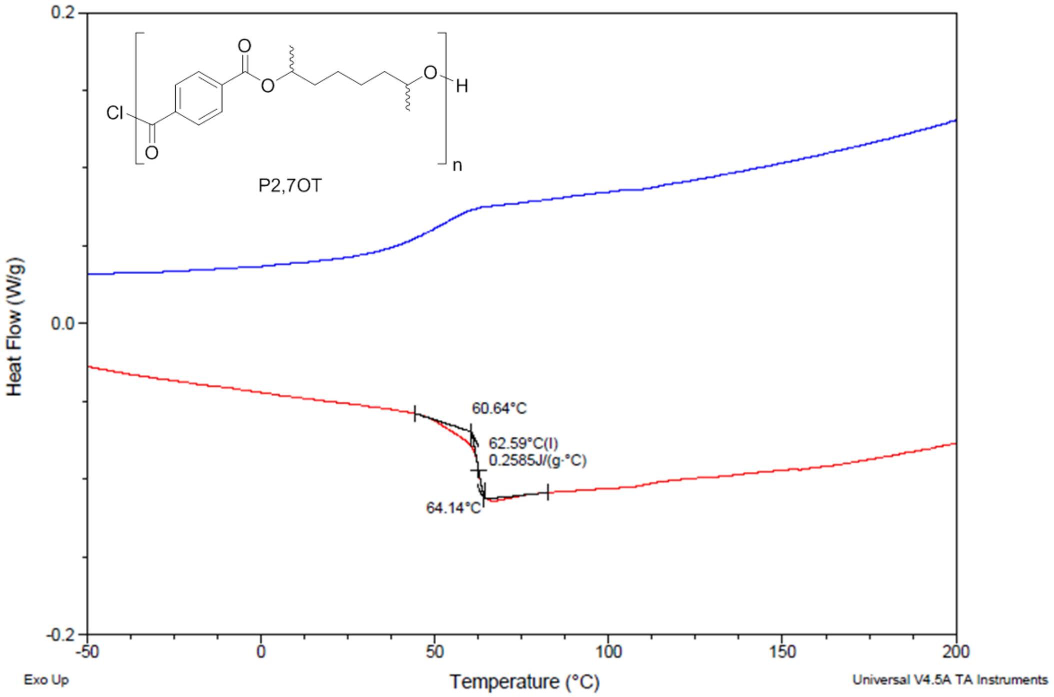

Figure S10. Modulated DSC trace of **4**.

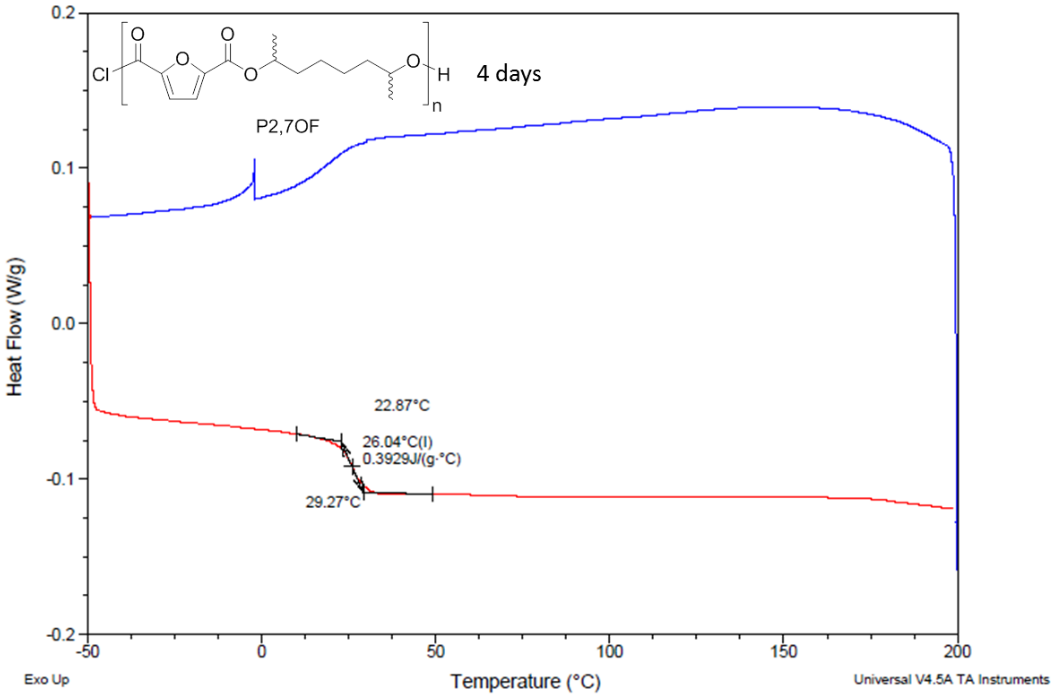

Figure S11. Modulated DSC trace of **5**.

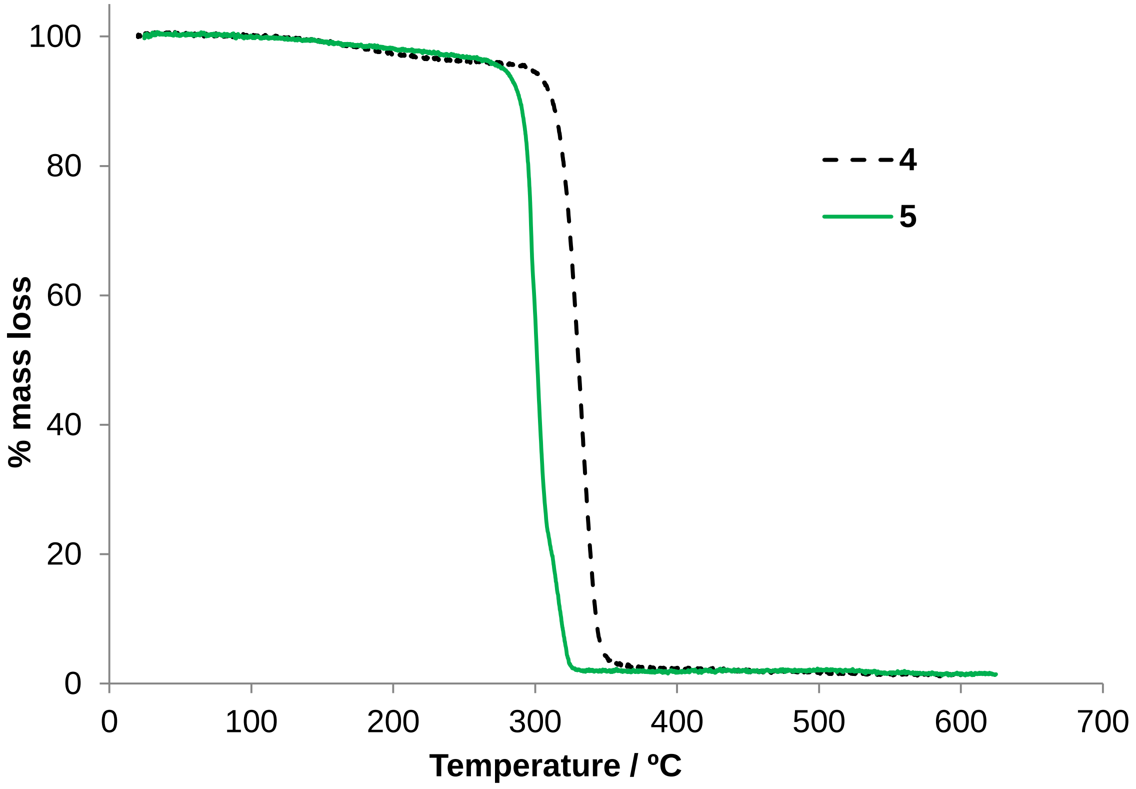

Figure S12. Thermogravimetric profiles for polymers **4** and **5**.

Table S3. Comparison of the glass transition temperature (T_g) of polyesters of 2,7-octanediol **3** with previously reported aliphatic-aromatic polyesters.

Diacid unit	Diol unit	T_g / °C	Source
Terephthalic acid (TA)	1,4-butanediol	41	[1]
	2,3-butanediol	127	[2]
	1,6-hexanediol	6	[3]
	1,8-octanediol	5	[3]
	2,7-octanediol	63	This work
2,5-furandicarboxylic acid (FDCA)	1,4-butanediol	26-36	[4]
	2,3-butanediol	87	[5]
	1,6-hexanediol	12-16	[4]
	1,8-octanediol	6-13	[4]
	2,7-octanediol	26	This work

References

[1] M. Pyda, E. Nowak-Pyda, J. Heeg, H. Huth, A. A. Minakov, M. L. Di Lorenzo, C. Schick, B. Wunderlich, *J. Polym. Sci., Part B: Polym. Phys.* **2006**, *44*, 1364-1377.

[2] W. J. Jackson, J. J. Watkins, US 4,600,768, 1986.

[3] T. H. Ng, H. L. Williams, *Makromol. Chem*. **1981**, *182*, 3323-3330.

[4] Y. Jiang, A. J. J. Woortman, G. O.R. Alberda van Ekenstein, K. Loos, *Polym. Chem*. **2015**, *6*, 5198-5211.

[5] S. Thiyagarajan, W. Vogelzang, R. J. I. Knoop, A. E. Frissen, J. van Haveren, D. S. van Es, *Green Chem.* **2014**, *16*, 1957-1966.
